# F-Spondin Deficient Mice Have a High Bone Mass Phenotype

**DOI:** 10.1371/journal.pone.0098388

**Published:** 2014-05-29

**Authors:** Glyn D. Palmer, Mukundan G. Attur, Qing Yang, James Liu, Paxton Moon, Frank Beier, Steven B. Abramson

**Affiliations:** 1 Division of Rheumatology, New York University School of Medicine and NYU Hospital for Joint Diseases, New York, New York, United States of America; 2 Department of Physiology and Pharmacology, Schulich School of Medicine and Dentistry, The University of Western Ontario, London, Ontario, Canada; INSERM U1059/LBTO, Université Jean Monnet, France

## Abstract

F-spondin is a pericellular matrix protein upregulated in developing growth plate cartilage and articular cartilage during osteoarthritis. To address its function in bone and cartilage *in vivo*, we generated mice that were deficient for the F-spondin gene, *Spon1*. *Spon1* **^−^** ^*/*−^ mice were viable and developed normally to adulthood with no major skeletal abnormalities. At 6 months, femurs and tibiae of *Spon1* **^−^** ^*/*−^ mice exhibited increased bone mass, evidenced by histological staining and micro CT analyses, which persisted up to 12 months. In contrast, no major abnormalities were observed in articular cartilage at any age group. Immunohistochemical staining of femurs and tibiae revealed increased levels of periostin, alkaline phosphate and tartrate resistant acid phosphatase (TRAP) activity in the growth plate region of *Spon1* **^−^** ^*/*−^ mice, suggesting elevated bone synthesis and turnover. However, there were no differences in serum levels of TRAP, the bone resorption marker, CTX-1, or osteoclast differentiation potential between genotypes. Knockout mice also exhibited reduced levels of TGF-β1 in serum and cultured costal chondrocytes relative to wild type. This was accompanied by increased levels of the BMP-regulatory SMADs, P-SMAD1/5 in tibiae and chondrocytes. Our findings indicate a previously unrecognized role for *Spon1* as a negative regulator of bone mass. We speculate that *Spon1* deletion leads to a local and systemic reduction of TGF-β levels resulting in increased BMP signaling and increased bone deposition in adult mice.

## Introduction

F-spondin (also known as SPON1 and VSGP) is a secreted, heparin-binding extracellular matrix glycoprotein that belongs to the thrombospondin (TSR) family. It is primarily associated with the regulation of neuronal outgrowth in the embryonic central nervous system [1]–[4]. More recently, F-spondin expression has been found in non-neuronal tissues including ovary, lung [5], periodontal tissue [6], embryonic growth plate cartilage [7] and osteoarthritic cartilage [8]. Despite these observations, precise physiological roles for F-spondin have yet to be elucidated.

*In vitro* studies have shown that F-spondin exhibits diverse cell-regulatory functions. The C-terminal thrombospondin type 1 repeat (TSR) domain of the protein has been shown to mediate many of its biological activities, including inhibition as well as promotion of axonal outgrowth [2], [4] regulation of amyloid precursor protein cleavage [9], induction of PGE2 [8], ECM binding [3] and cell survival [10]. The TSR domain also harbors highly conserved motifs, KRFK and WxxW, which have been shown to be required for thrombospondin mediated activation of the latent TGF-β complex [11]. Early evidence suggests that the F-spondin TSR also operates as a functional TGF-β activation domain. Treatment of human cartilage explant cultures with F-spondin was found to increase active TGF-β levels [8], while the addition of the TSR domain to chick ciliary ganglion neuron cultures has been shown to substitute the prosurvival effects of TGF-β [10].

Beyond CNS regulation, several *in vitro* studies also implicate F-spondin in the regulation of musculoskeletal tissues. In periodontal tissue, F-spondin localizes to mineralized cementum and has been shown to promote osteoblast-like differentiation of periodontal ligament cells into cementoblasts via induction of BMP-7 [6], [12]. It has also been shown to inhibit M-CSF induced cell migration and differentiation of osteoclasts, in RAW 264 cell lines, suggesting an additional anti-resorptive role in this tissue [13]. In articular cartilage, we have previously reported induction of F-spondin expression in human and rodent osteoarthritis [14]. In *ex vivo* cultures of embryonic mouse tibiae, we showed inhibition of longitudinal growth and altered growth plate morphology following exogenous F-spondin addition, providing the strongest indication yet, that F-spondin is involved in skeletal development [7]. Despite these observations, the lack of gene knockout studies prevents a more definitive assessment of the role of F-spondin in bone and cartilage physiology.

To address this further, the aim of the present study was to identify the role of F-spondin in bone and cartilage development *in vivo*, in mice that are null for the F-spondin gene, *Spon1*. Despite our earlier findings, these mice undergo normal skeletal development, but show enhanced postnatal accumulation of trabecular and cortical bone in the femur and tibia. These changes are accompanied by abnormalities in components of the TGF-β signaling pathway including TGF-β1 and SMADs. This report represents the first indication for physiological control of bone metabolism by F-spondin, possibly via disruption of latent TGF-β activation.

## Materials and Methods

### Generation of *Spon1* ^−*/*−^ mice and genotyping

**Figure 1:**
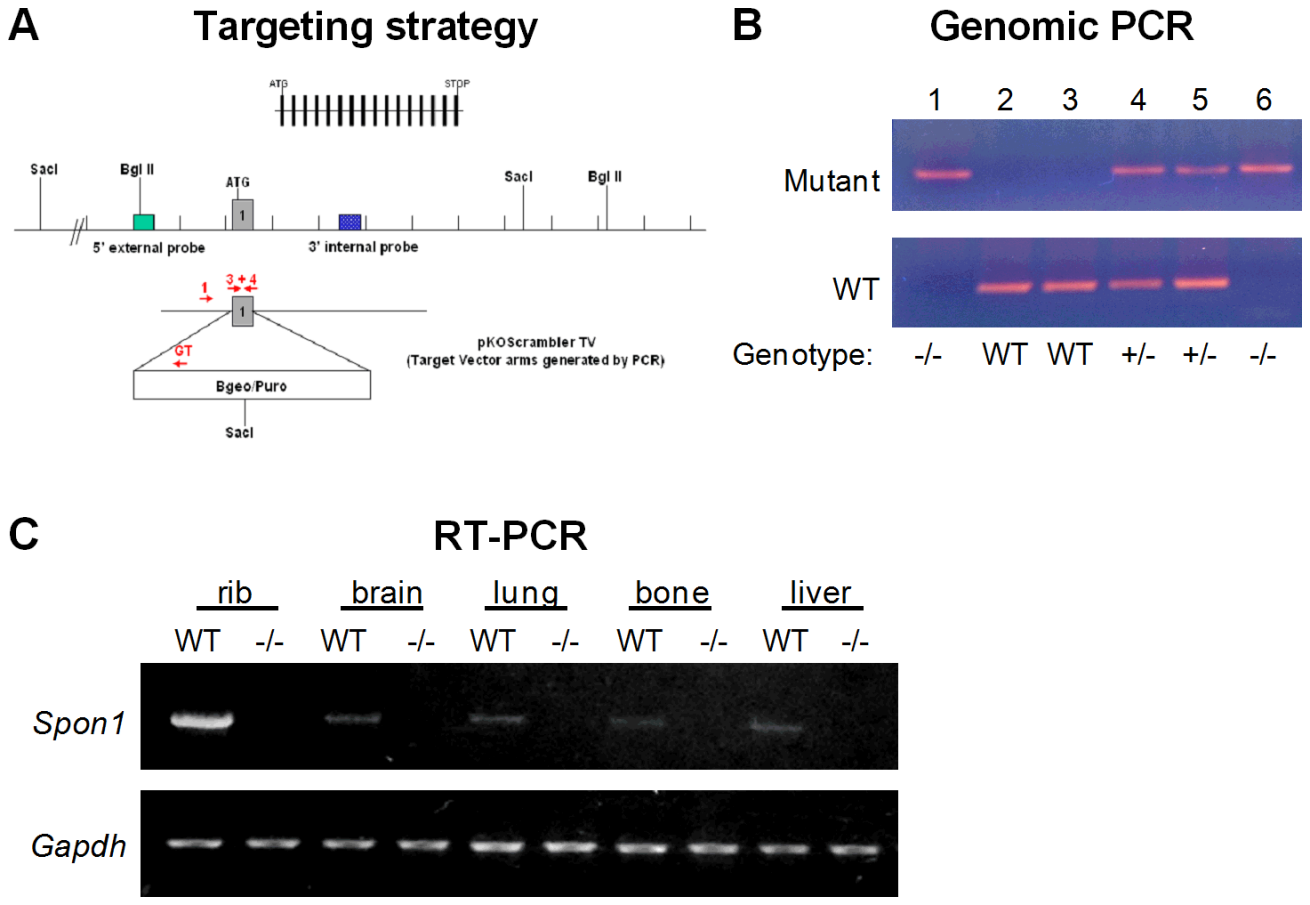
Targeting strategy for generation of *Spon1* ^−/−^ mice. (A) The murine *Spon1* gene was disrupted by homologous recombination to replace exon 1 (represented by grey box) with an IRES/bGeo/PolyA cassette. Numbered arrows (red) indicate PCR primers used for genotyping of WT and mutant loci. (B) Identification of *Spon1* mutant mice by PCR. Wild type (WT) mice were distinguished by PCR products from primer set 3 + 4, null mice (−/−) were positive for 1 + GT, and heterozygous mice (+/−) were positive for both. (C) RT-PCR analysis of *Spon1* expression in selected tissues. *Gapdh* amplification was performed as a housekeeping control.

*Spon1* **^−^** ^/−^ mice were generated in collaboration with the Texas Institute of Genomic Medicine (TIGM) using a gene targeting strategy that replaces the first coding exon of *Spon1* with an IRES/bGeo/PolyA cassette via homologous recombination. This recombination removes the ATG start codon, preventing translation initiation. Mutant ES clones were generated and confirmed by Southern blotting with 5' and 3′ flanking probes. Chimeric mice were then generated by injection of ES cells into blastocysts of C57BL/6 mice. *Spon1* **^−^^/−^** mice were obtained by crossing heterozygous progeny of chimeric mice. Genotyping was performed using genomic DNA isolated from mouse tail biopsies using allele-specific primers as follows: Primer 3, GACCGGAGATCTAGGAACCCCTAG, and primer 4, CACTCTCGCCAACAGCTGGAGCG, were designed from the first intron and used to amplify the wild type allele, generating a 318-bp PCR product; Primer 1, CTCCGCTCAGAGCAGCGCAGCTC, and primer GT, CCCTAGGAATGCTCGTCAAGA, were designed from the targeting vector and used to detect the mutant allele, producing a 391-bp PCR product. The *Spon1* targeting and genotyping strategy are summarized in Figure 1A. For all analyses, mice were euthanized by cervical dislocation following anesthesia with xyaline and ketamine in accordance with the guidelines of the Institutional Animal Care and Use Committee (IACUC) of New York University.

### Ethics Statement

This study was carried out in strict accordance with the recommendations in the Guide for the Care and Use of Laboratory Animals of the National Institutes of Health. All procedures performed in the study were approved by the Institutional Animal Care and Use Committee (IACUC) of New York University School of Medicine under protocol number 121006-01.

### RT-PCR analysis

Tissues (brain, bone, lung, liver) were harvested from 6 month old male mice or 5 day old littermates (rib cartilage) and homogenized using a tissue grinder. For bone, whole tissue including matrix, bone marrow and periosteum was processed for RNA extraction. Total RNA was extracted using Trizol reagent and RNA was purified using an RNeasy micro kit (Qiagen) according to the RNeasy cleanup protocol. cDNA was synthesized from 1 µg of RNA using MMLV reverse transcriptase and Oligo(dT)_18_ as primers (Clontech). PCR was performed using *Spon1* specific primers AGAGGCACCGTATGGTCAAG (forward) and GACCACTCGCTGAGTTCACA (reverse). *Gapdh* (Clontech, #ST0253) was used as a housekeeping control. After amplification, products were resolved on a 1% agarose gel and visualized by ethidium bromide staining.

### Histological and immunohistochemical analyses

Whole embryo staining, dissection and paraffin embedding of long bones, preparation of paraffin sections and safranin O/Fast Green staining was performed as described [15]. Growth plate measurements were performed by a blinded observer as before [16].

For adult mice, knee joints were dissected from 6 month old males and fixed for four days in 4% paraformaldehyde in PBS at 4°C. Specimens were decalcified in 10% EDTA in 0.05 M Tris buffer at RT, until bones were soft and flexible (4–7 days at RT), dehydrated in an ethanol series, cleared in xylene and embedded in paraffin. Sections were cut to 5 µm and stained with Weigert's hematoxylin/Fast Green/Safranin-O for bone and cartilage. Immunohistochemistry was performed at the histology core of Hospital for Special Surgery, using antibodies for F-spondin (rabbit polyclonal R4 antibody), alkaline phosphatase (Abcam), and periostin (Sigma). All of these primary antibodies were localized by the ABC avidin-streptavidin kit (Vector Laboratories) resulting in a brown reaction product. All tissues were counterstained with hematoxylin. For TRAP staining sections were reactivated in 0.2 M Tris buffer and incubated with TRAP medium for 1–2 h at RT according to the method of Liu et al. [17] and Barka and Anderson [18]. Sections were counterstained with 1% methyl green. Quantification of cartilage area and positive immunostaining was performed using image J software (http://rsbweb.nih.gov/). For each sample values represent an average of a minimum of 4 stained sections of equivalent depth.

### Micro CT analyses

Mouse knees were studied by micro-computed tomography (µCT) on Scanco mCT 35 (Scanco Medical, Bassersdorf, Switzerland) system. Scans were performed in DPBS using 6 µm voxel size, 55KVp, 0.36 degrees rotation step (180 degrees angular range) and a 400 ms exposure per view. For trabecular bone, micro CT evaluation was performed on a 1.2 mm region of metaphyseal spongiosa in the proximal tibia and distal femur. The regions were located 100 microns above (femur) or below (tibia) the growth plate. For cortical bone, measurements were performed on a 1.4 mm region of the mid-diaphysis of the femur. The Scanco µCT software (HP, DECwindows Motif 1.6) was used for 3D reconstruction and viewing of images. After 3D reconstruction, volumes were segmented using a threshold of 0.4 g/c for trabecular and 0.6 g/cc for cortical bone. Tissue mineral density (TMD) and directly measured bone volume fraction (BV/TV), surface to volume ratio (BS/BV), thickness (Tb.Th), number (Tb.N) and separation (Tb.Sp) were calculated for the trabecular bone. Cortical bone measurements included total area (TA), bone area (BA), porosity, and thickness of the cortex (CtTh). Only male mice (WT and KO) were included for µCT analyses.

### Serum Biomarker Assays

Serum samples were collected from 6 and 12 month old WT and KO mice and assayed for the bone degradation markers, CTX-1, and TRAP (TRACP 5b) using commercially available ELISA kits (Immune Diagnostic System Inc Fountain Hill, AZ).

### Cell culture

Chondrocytes were isolated from intact rib cages harvested from 5 d old littermates according to the method of Gosset M et al. [19]. Cells were cultured to confluence (4–5 days after harvest) in DMEM with 10% FBS. Upon confluence, the media was changed and ascorbate 2-phosphate (50 µg/ml) and β-glycerolphosphate (10 mM) were added to initate hypertrophic differentiation. After 48 h, media was collected for measurement of TGF-β levels, and cells were lysed for detection of phosphorylated SMADs.

### Osteoclast Differentiation

Bone marrow was flushed from cut femurs and tibiae with a syringe containing αMEM supplemented with 10% FCS and plated overnight. Non-adherent cells were collected the following day and purified by Ficoll gradient centrifugation. Collected cells were washed and resuspended in osteoclast induction medium (αMEM containing 10% FCS, 40 ng/ml M-CSF and 60 ng/ml RANKL). Control cultures (day 0) contained media without cytokine supplementation. After 6 days, cells were fixed with citrate solution and stained for TRAP using the Acid Phosphatase, Leukocyte (TRAP) Kit and protocol (Sigma). For quantitative gene expression analysis, RNA was extracted at various time points, reverse transcribed and real-time PCR reactions were performed with SYBR green PCR reagents using the ABI Prism 7300 sequence detection system (Applied Biosystems).

### Measurement of TGF-β levels

To determine TGF-β levels in recovered tissues of wild type and *Spon1* **^−^** ^/−^ mice, specimens were lysed in RIPA lysis buffer. Total TGF-β1 levels were then measured by ELISA (R&D systems) following acidification of samples by treatment with 0.2 M HCl according to the manufacturer's protocol. TGF-β1 levels were normalized to total protein content for each sample, determined using the BCA protein assay kit (Pierce). In costal chondrocyte cultures, total TGF-β1 levels were measured by ELISA following acidification of cell culture supernatants. For accurate quantification of active TGF-β levels, we employed a cell-based reporter assay as previously described [8]. Briefly, mink lung epithelial cells (MLEC), stably transfected with a TGF-β responsive luciferase reporter gene, were incubated overnight with chondrocyte culture media supernatants and assayed for luciferase activity using the Luciferase reporter assay system (Promega). Luminescence was measured using a Tristar LB 941 luminometer (Berthold Technologies).

### Detection of SMADs

Proteins were extracted from bone tissue specimens and chondrocytes by incubation with RIPA lysis buffer (Santa Cruz) for 30 min at 4°C. After centrifugation, proteins were quantified using BCA reagent and 50 µg of cell lysates were electrophoresed on a 10% SDS-PAGE gel. Proteins were transferred to polyvinylidene difluoride membranes (Millipore Corporation) and blots were probed with monoclonal antibodies against phosphorylated and non-phosphorylated SMADs from the Phospho-SMAD Antibody Sampler Kit (Cell Signaling Technologies). β-tubulin was used as loading control. Horseradish peroxidase-coupled secondary antibodies were used for detection (Jackson Immuno Research Laboratories) and blots were developed using the enhanced chemiluminescence Western blot system (Pierce) on a Kodak automated developer. Semi-quantitative measurements of SMAD levels were performed by denistometric analysis of immunoblots using image J software.

### Statistics

Results are expressed as mean ± SEM. For µCT, TGF-β and SMAD measurements, statistical differences between groups were assessed by students *t* test. *P*<0.05 was taken as minimum significance level.

## Results

### *Spon1* ^−*/*−^ mice are viable and show histological evidence of increased bone

F-spondin *(Spon1* **^−^** ^/−^) null mice were generated using a targeting strategy that removes the first exon of *Spon1* (Fig 1A), and genotyped using mutant and allele-specific primers (Fig 1B). All mice were viable and born at the expected Mendelian ratio of 1∶2∶1 (wild type:heterozygous:null). Initial macroscopic observation at birth and in mice aged 1-12 months revealed no gross anatomical differences or significant changes in body weight between wild type, heterozygous, and null mice. In wild type mice (WT), RT-PCR revealed expression of F-spondin mRNA in brain, lung, bone, liver and hypertrophic cartilage from rib, consistent with our previous observations (Fig 1C) [7]. Among isolated cell cultures of growth plate chondrocytes, calvaria osteoblasts, and in vitro differentiated osteoclasts, *Spon1* expression was detected only in chondrocytes (data not shown), in agreement with our previous analysis of chick and mouse cartilage [7]. Importantly, no *Spon1* PCR products were detected in any tissues or cell cultures isolated from knockout (KO) mice (Fig 1C).

**Figure 2:**
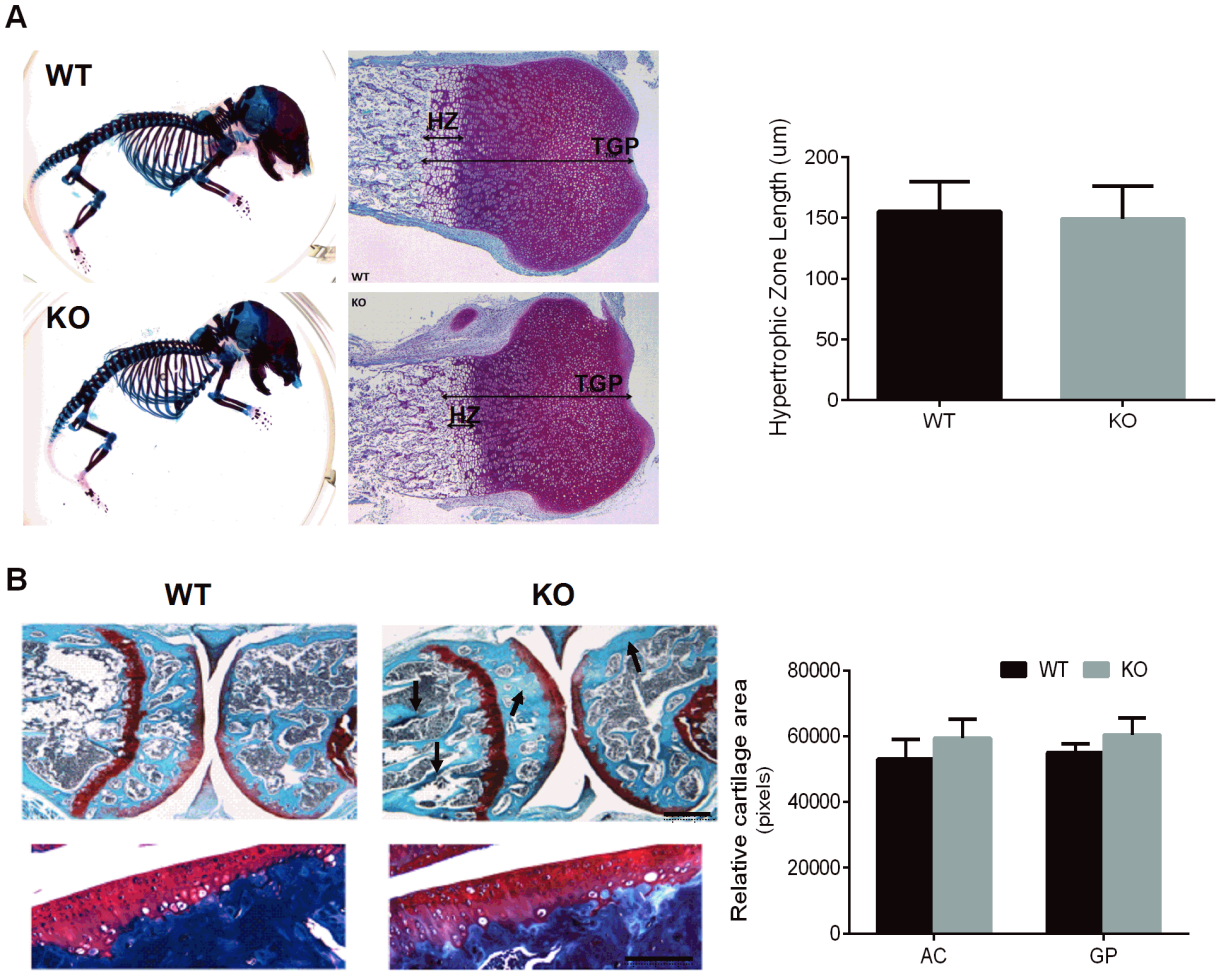
*Spon1* ^−/−^ mice exhibit post-natal accumulation of bone. (A) WT and *Spon1* **^−^** ^*/*−^ skeletons were prepared from newborn (P0) littermates and stained with Alizarin Red/Alcian Blue to assess skeletal size and ossification. *Right Panel* P0 Tibiae were stained with H&E and the areas corresponding to the hypertrophic zone (HZ) and total growth plate (TGP) measured. Graph shows similar average HZ lengths in *Spon1* **^−^** ^/**−**^ and WT from 6 littermate pairs. (B) Safranin O/Fast Green staining of tibia and femur from 6 month old male mice. Panels below show Safranin-O/Fast Green staining articular cartilage from the medial tibial plateau of 6 month old male mice. Arrows denote areas of increased bone deposition in *Spon1* **^−^** ^/−^ mice. Graph indicates mean area of tibial articular cartilage (AC) and growth plate cartilage (GP) for 5 WT and KO mice; differences were not significant (*P*≥0.2). Scale bars; 200 µm for knee sections and 100 µm for articular cartilage.

Since *Spon1* expression has previously been demonstrated in embryonic growth plate chondrocytes and osteoarthritic cartilage, we first examined neonatal and adult mice for evidence of skeletal malformations and possible cartilage/bone phenotypes. Whole body skeletal staining of newborn mice by Alizan Red (bone) and Alcian Blue (cartilage) demonstrated no abnormalities in skeletal size or morphology between genotypes (Fig 2A). Histological analyses of sections of long bones from WT and *Spon1* **^−^** ^/−^ mice at birth also revealed no differences in bone length, ossification patterns or hypertrophic zone length in the growth plate (Fig 2A). Thus despite its reported expression in embryonic cartilage, *Spon1* deletion did not appear to impair growth plate maturation or skeletal formation. In contrast, histological analysis of mouse joints at 6 months suggests involvement of *Spon1* in the regulation of bone. (Fig 2B). Safranin-O/Fast Green staining revealed a marked increase in trabecular bone staining in *Spon1* **^−^** ^/−^ mice in the medullary cavity and epiphyseal region below the articular cartilage (*arrows*, Fig 2B), while the areas of articular and growth plate cartilage were similar between genotypes (*graph*, Fig 2B). These observations suggest a bone, but not cartilage phenotype in *Spon1* **^−^** ^/−^ mice.

### *Spon1* ^−*/*−^ mice shave increased trabecular and cortical bone by micro-CT

**Figure 3:**
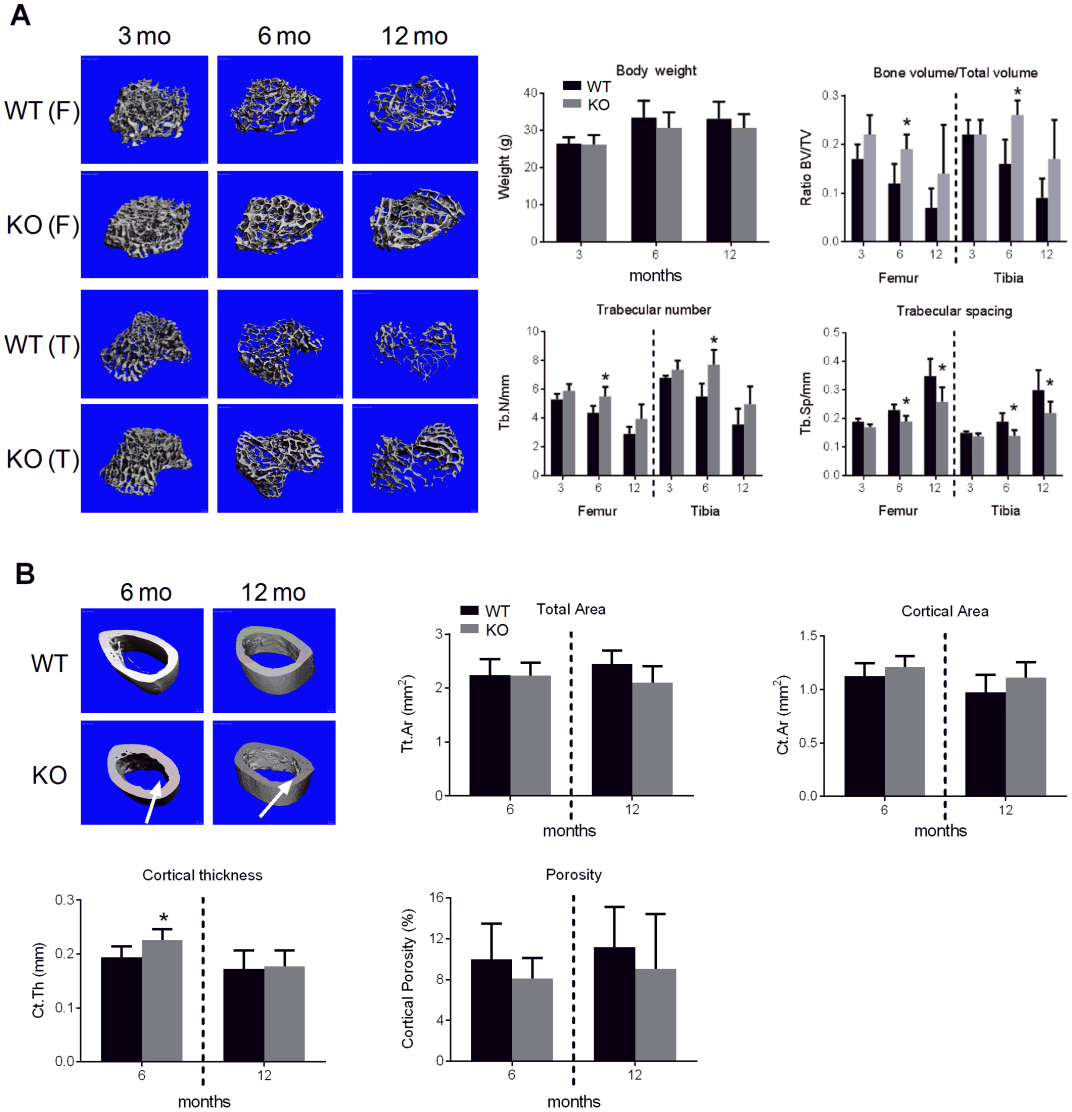
*Spon1* ^−/−^ mice have increased trabecular and cortical bone. (A) Representative 3D µCT images showing regions of trabecular bone in the distal femur (F) and proximal tibia (T). Graphs represent measurements of total body weight (g), trabecular bone volume (bone volume per tissue volume, BV/TV), trabecular number (Tb.N/mm) and trabecular spacing (Tb.sp/mm) in the femurs and tibiae of 3 month (n = 4), 6 month (n = 10–11) and 12 month (n = 6) -old *Spon1* **^−^** ^/−^ and WT mice. (B) Representative 3D µCT images showing regions of femoral cortical bone in WT and *Spon1* **^−^** ^/−^ mice. Graph represents measurement of total area (Tt.Ar mm^2^), cortical area (Ct.Ar mm^2^, cortical thickness (Ct.Th/mm) and intracortical porosity (%) of 6 month (n = 6–11) and 12 month (n = 5) mice. Arrows show the irregular surfaces along the endosteal perimeters of *Spon1* **^−^** ^*/*−^ mice. Statistically significant differences from WT mice groups are indicated by *(*P*<0.05).

To establish whether increased trabecular staining reflects a high bone mass condition in *Spon1* **^−^** ^/−^ mice, we performed quantitative analyses of bone structure using micro-CT on femurs and tibiae from mice aged 3, 6 and 12 months. Relative to WT, femurs and tibiae from *Spon1* **^−^** ^/−^ mice exhibited a marked increase in trabecular bone (Fig 3A). At 6 months, this phenotype was most pronounced, evidenced by a ∼60% higher bone volume (Fig 3A; *P*<0.001). This was accompanied by increased femur and tibia trabecular number (26 and 40% respectively; *P*<0.0005) and decreased trabecular spacing (Tb.Sp) (18 and 26%; *P*<0.01). In contrast, there were no differences in either trabecular thickness (Tb.Th) or total mineral density (TMD). Increased trabecular bone was still apparent at 12 months, however only Tb.Sp remained statistically significant (*P*<0.05). Micro-CT measurements of cortical bone revealed a trend of increased cortical area and decreased porosity at both 6 and 12 months in *Spon1* **^−^** ^/−^ mice; however these differences were not significant (Fig 3B). *Spon1* **^−^** ^*/*−^ mice also had increased femurs cortical thickness (∼16%, *P*<0.01) relative to WT mice at 6 months, but this was not evident at 12 months (Fig 3B). Interestingly, 3D micro-CT images revealed that the endocortical perimeter of *Spon1* **^−^** ^/−^ femurs appeared distinctly irregular at both 6 and 12 months, suggesting abnormalities in bone remodeling (arrows, Fig 3B). Together the data indicate a high bone mass phenotype in femurs and tibiae of *Spon1* **^−^** ^/−^ mice; the phenotype appears to be age-dependent and most pronounced at 6 months of age.

### *Spon1* ^−*/*−^ mice have increased levels of bone markers

**Figure 4:**
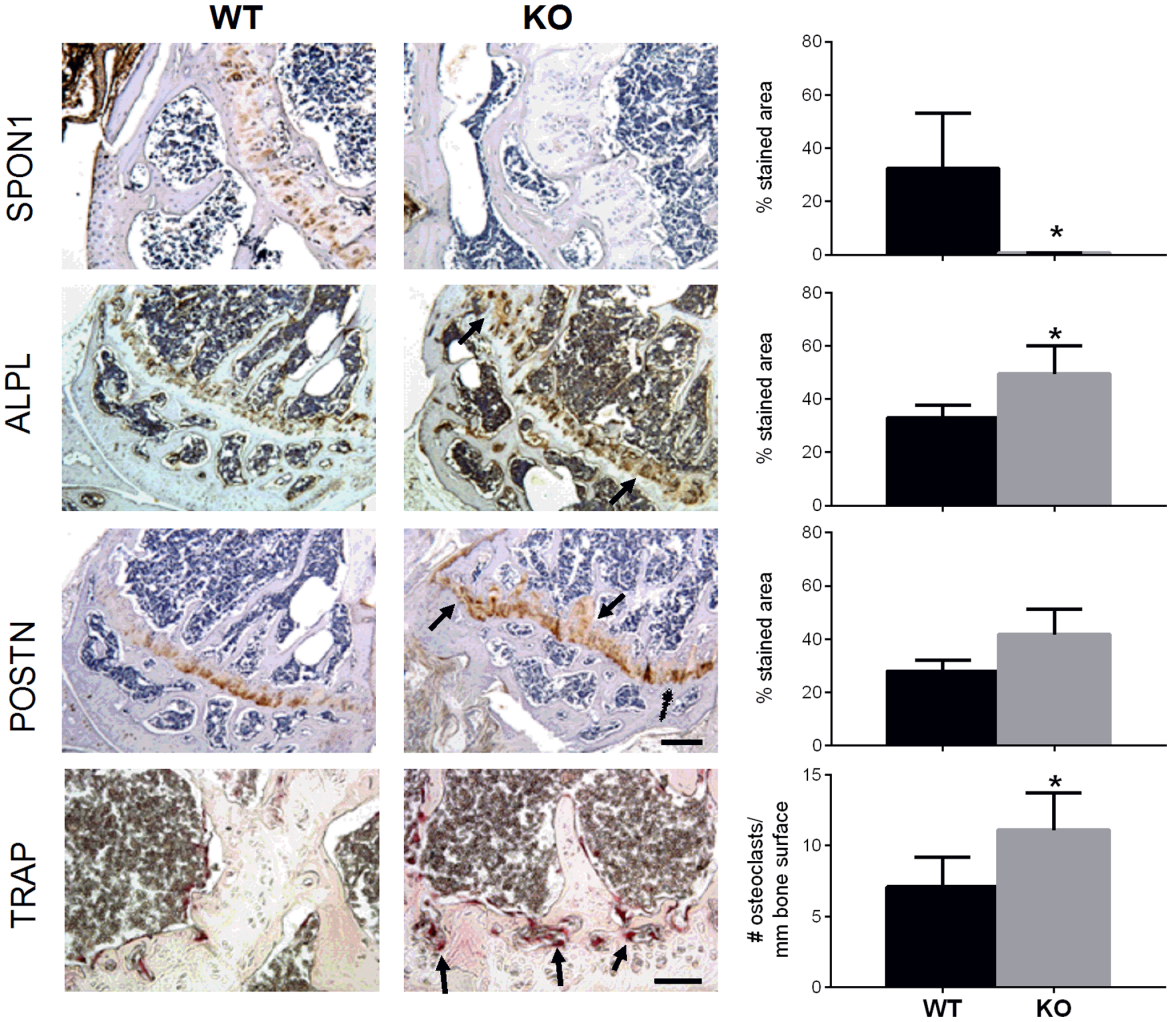
*Spon1* ^−/−^ mice tibiae exhibit increased staining of markers of bone synthesis and turnover. Immunohistochemical staining of mid-saggital sections of tibia from 6 month old WT and *Spon1* **^−^** ^*/*−^ mice with antibodies for F-spondin (SPON1), Alkaline Phosphatase (ALPL), Periostin (POSTN), and tartrate resistant acid phosphatase (TRAP) as a marker of osteoclast activity. Sections were counterstained with either 1% methyl green (TRAP) or hematoxylin. Arrrows indicate areas of increased staining in the epiphyseal cartilage and adjacent trabecular bone of *Spon1* **^−^** ^/−^ tibiae. Adjacent graphs correspond to quantification of positive stained area (SPON1, ALP, POSTN) and osteoclast number per field (TRAP). Quantification was performed on 4 sections per specimen, and values represent the average of 4 mice per genotype. * = *P*<0.05. Scale bars; 200 µm for antibody stainings and 100 µm for TRAP staining.

We next determined whether the differences in bone architecture were accompanied by correlative changes in markers of bone formation and resorption. To address this we performed immunostaining of 6 month old tibiae for markers associated with bone turnover. Staining of 6 month old tibiae with anti-F-spondin antibody revealed positive cell- and matrix-associated staining in epiphyseal cartilage of WT mice (Fig 4). The corresponding region in *Spon1* **^−^** ^/−^ mice was negative for F-spondin staining, but revealed greater levels of bone markers associated with increased synthesis and turnover. Along the growth plate and adjacent trabecular bone of KO mice, the relative area of positive immunostaining was 1.5-fold higher for alkaline phosphatase and periostin compared to WT (Fig 4). Somewhat contradictory to the high bone mass phenotype, this region also had increased numbers of TRAP positive cells, mostly concentrated at the junction of the epiphyseal growth plate and bone marrow cavity (Fig 4). The same pattern was observed in the femurs (not shown). Together these findings indicate that the absence of *Spon1* in epiphsyeal cartilage associates with increased levels of bone synthesis and resorption markers in the growth plate and adjacent trabecular bone.

### *Spon1* deletion does not affect serum biomarker levels or osteoclast differentiation

**Figure 5:**
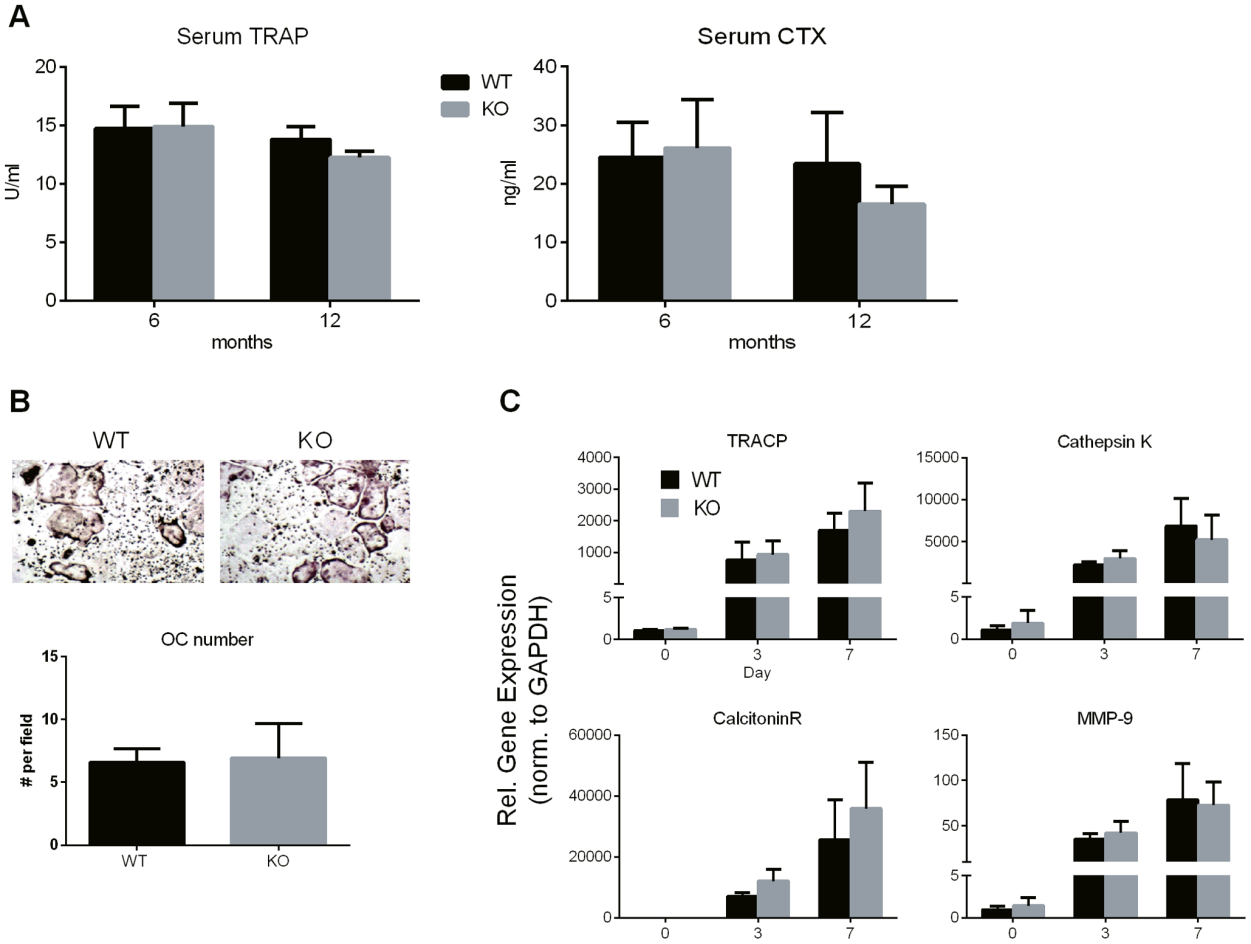
Biomarkers of bone resorption and osteoclast differentiation potential are not affected by *Spon1* ^−^^*/*−^ deletion. (A) Serum levels of TRAP and CTX-1 in 6 month (n = 12) and 12 month (n = 6) old male mice. (B) *In vitro* osteoclast differentiation of non-adherent bone marrow cells isolated from male WT and KO littermates. Images show TRAP positive osteoclasts 6 days after stimulation with RANKL and MCSF. Cell numbers represent average counts from 5 separate fields using a 20x objective (n = 4). Only positively stained cells with >3 nuclei were counted. (C) Relative mRNA levels, determined by RT-qPCR of osteoclast marker genes, following induction of differentiation (n = 4). Data represent mean values from 4 littermate pairs.

The elevated TRAP staining in mouse tibiae of *Spon1* **^−^** ^/−^ mice was surprising given that the mice exhibit an increased bone mass phenotype. Therefore, we examined serum markers of bone resorption for further evidence of enhanced bone turnover in *Spon1* **^−^** ^/−^ mice. Serum levels of TRAP and CTX-1 - a C-terminal telopeptide of type I collagen, were measured in 6 and 12 month old WT and KO mice. Levels of both markers did not significantly change in KO mice relative to WT for either age group indicating that overall resorption is in fact similar between genotypes (Fig 5A).

We next determined whether F-spondin deletion in bone marrow progenitor cells leads to an enhanced ability for osteoclast maturation and could thus account for increased TRAP staining in *Spon1* **^−^** ^/−^ tibiae. Bone marrow was harvested from 6 month old WT and *Spon1* **^−^** ^/−^ littermates and non-adherent fraction was subjected to M-CSF and RANKL induced differentiation, *ex vivo*. After 6 days of induction, numbers of TRAP positive osteoclasts were counted and compared between genotypes. No differences were observed in osteoclast number between WT and KO mice (Fig 5B). Similarly, expression of mRNAs for osteoclast marker genes, acid phosphatase-5, cathepsin K, calcitonin receptor and *MMP9* did not significantly change in KO mice (Fig 5C). Thus, despite the high bone mass condition indicated by micro-CT, and a localized increase of TRAP positive cells along the growth plate, no further indication of abnormalities in bone turnover or osteoclast activity were noted in response to *Spon1* deletion.

### *Spon1* ^−*/*−^ mice have reduced levels of serum and chondrocyte-derived TGF-β

**Figure 6:**
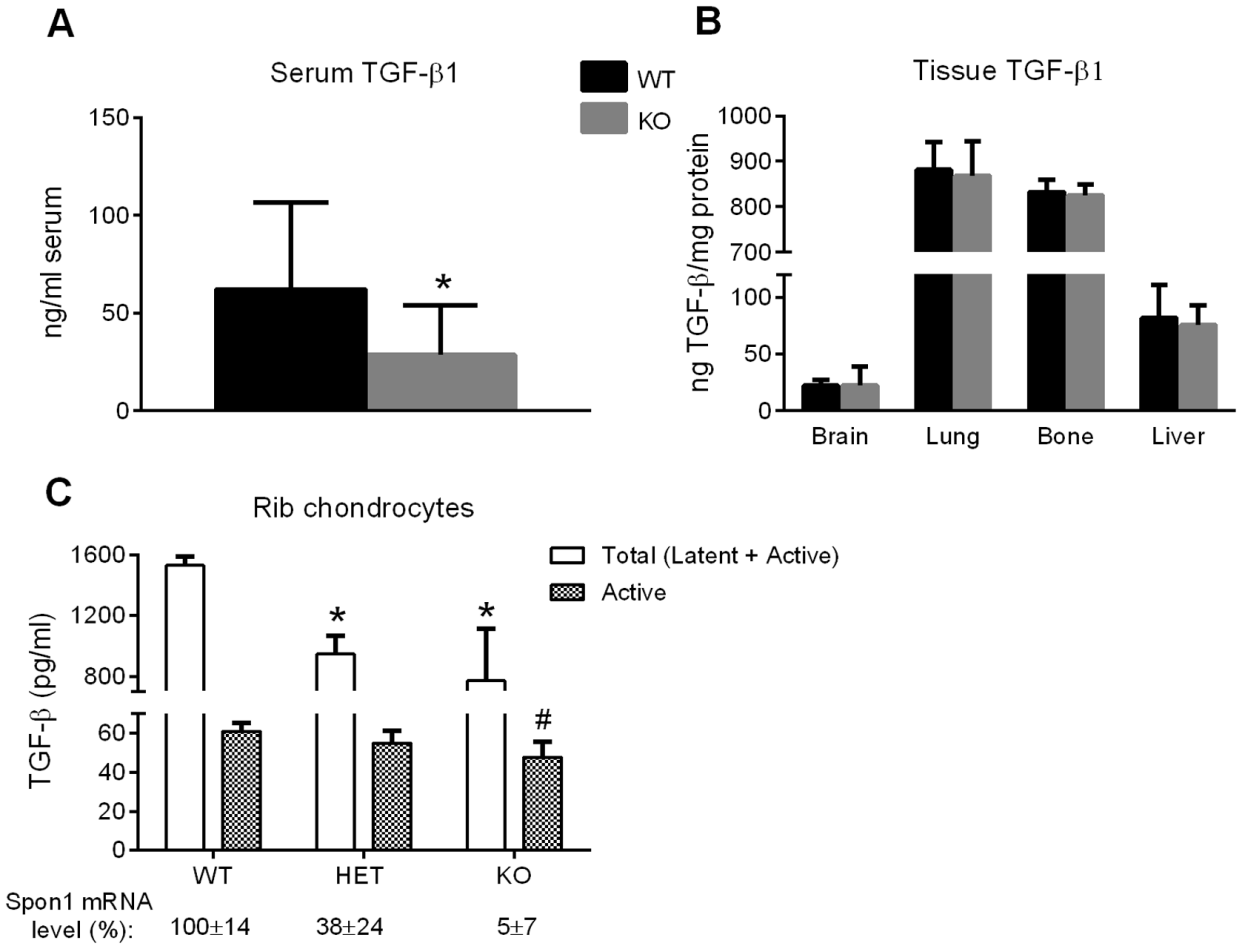
TGF-β1 levels are reduced in serum and chondrocytes of Spon1^−/−^ mice. Total (latent + active) TGF-β1 levels in protein extracts isolated from (A) serum (n = 14-16) or (B) indicated organs (n = 4–5) of 6 month old WT and *Spon1* **^−^** ^/−^ mice. TGF-β1 was measured by ELISA. (C) Total and active TGF-β levels in conditioned media of rib chondrocyte cultures (n = 4) isolated from costal cartilage of 5 day old WT, HET and KO littermates. Total TGF-β levels were determined by ELISA, active TGF-β levels were measured using MLEC bioassays. Corresponding *Spon1* mRNA levels, determined by QPCR, are shown for each group (WT = 100%). Statistically significant differences in total TGF-β level to WT group are indicated by *(*P*<0.05). Statistically significant difference in active TGF-β level to WT group is indicated by # (*P*<0.05).

TGF-β is known to regulate multiple aspects of bone synthesis and resorption [20]. Our previous studies have demonstrated that F-spondin has the capacity to act as a latent TGF-β1 activating protein [8], therefore we next examined *Spon1* **^−^** ^/−^ mice for TGF-β1 levels, to determine whether the high bone mass condition in KO mice associates with abnormal TGF-β signaling. In serum, which contains a large pool of latent TGF-β1, total TGF-β1 levels were ∼50% lower in KO mice relative to WT (Fig 6A; *P*<0.05). However, among tissues that were positive for *Spon1* expression in WT mice by RT-PCR (see Fig 1B), brain, bone, lung and liver did not have significantly lower TGF-β1 levels following *Spon1* deletion (Fig 6B).

To examine more closely a direct link between *Spon1* deletion and TGF-β activation, we measured cell-derived TGF-β levels in *Spon1* **^−^** ^/−^ and WT mice. For this study we used hypertrophic chondrocytes since our expression data indicated high levels endogenous F-spondin in rib (Fig 1) and tibial cartilage (Fig 4) by qPCR and immunostaining, respectively. Moreover, in isolated cell cultures mRNA levels of F-spondin were detected in hypertrophic chondrocytes, but not osteoblasts or osteoclasts (data not shown). Levels of secreted, total and active TGF-β were examined in cell cultures of rib chondrocytes isolated from 5 d old mice (Fig 6C). In culture, these cells are known to exhibit a hypertrophic phenotype, and mimic the cellular processes of terminal differentiation observed during growth plate maturation of long bones. For these studies, *Spon1* ^+/**−**^ heterozygous mice were included as an additional group to determine whether *Spon1*-mediated TGF-β effects were gene-dose dependent. Cultures from both heterozygous and KO mice secreted less total and active TGF-β relative to WT (Fig 6C). In KO mice, total and active levels were significantly reduced by 50% and 23% respectively (*P*<0.05, Fig 6C). In heterozygous mice, TGF-β levels were intermediate between WT and KO. As expected, TGF-β levels correlated with *Spon1* mRNA levels for each genotype, indicating a dose-dependent effect (Fig 6C). Altogether, these findings provide evidence that serum and chondrocyte-derived TGF-β are regulated by *Spon1 in vivo.* Moreover, chondrocyte-mediated regulation of TGF-β by *Spon1* suggests a possible direct link between F-spondin expression in epiphyseal cartilage of femurs and tibiae and the regulation of bone mass.

### P-SMAD levels are altered in *Spon1* ^−*/*−^ mice

**Figure 7:**
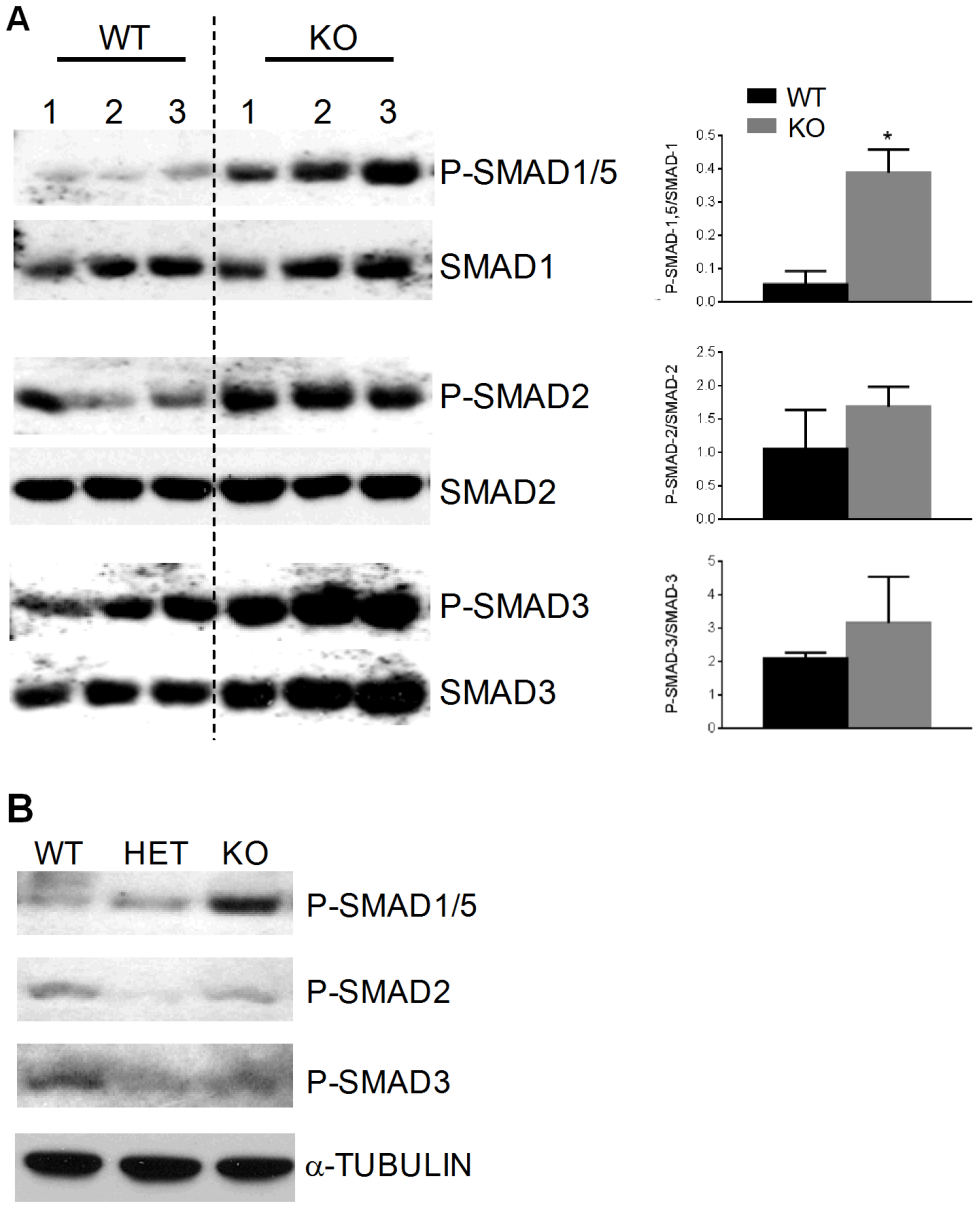
*Spon1* ^−^^*/*−^ mice have increased levels of P-SMAD1/5. (A) Western blot showing levels of SMAD and P-SMAD in protein extracts from whole tibia of 6 month old WT and *Spon1* **^−^** ^/−^ mice. Histograms represent densitometric analyses of SMAD blots. Statistically significant difference to WT group is indicated by *(*P*<0.001). (B) Western blot showing levels of P-SMAD in protein extracts from cultured costal chondrocytes isolated from 5 d old WT and *Spon1* **^−^** ^/−^ mouse rib cartilage. α-tubulin was used as a loading control. Blot is representative of chondrocytes isolated from 3 littermate pairs.

As further evidence of altered TGF-β canonical pathway signaling, we investigated levels of receptor regulated SMADs in WT and *Spon1* **^−^** ^*/*−^ mice. In both osteoblasts and maturing chondrocytes, SMAD2/3 activation is associated with TGF-β signaling, while SMAD1/5/8 is associated with BMP [21]–[24]. Western blot of protein extracts isolated from tibiae of 6 month old mice revealed that *Spon1* **^−^** ^/−^ mice showed a 7-fold increase in levels of Phosphorylated-SMAD1/5 (P-SMAD1/5) compared to WT (*P*<0.005; Fig 7A). In contrast, levels of P-SMAD2 and P-SMAD3 in KO mice were increased only marginally above WT controls (Fig 7A). To examine if *Spon1* deletion similarly affects P-SMAD levels in maturing chondrocytes, Western blot was also performed on extracts from parallel rib chondrocyte cultures from Fig 6B. Relative to WT, P-SMAD1/5 levels increased slightly in chondrocytes from heterozygous mice, and increased considerably in chondrocytes from KO mice (Fig 7B). In contrast, P-SMAD2 and P-SMAD3 levels were either unchanged or slightly lower after normalization (Fig 7B).

These findings show that *Spon1* KO mice have decreased TGF-β1 levels and enhanced activation of SMAD1/5 at both the tissue and chondrocyte level. The reduction in TGF-β1 levels may represent a shift towards BMP signaling in bone and hypertrophic cartilage, culminating in a high bone mass phenotype.

## Discussion

Our findings indicate that *Spon1* gene inactivation leads to increased bone in adult murine femurs and tibiae. This represents the first indication of the involvement of *Spon1* in the regulation of bone metabolism. Bone changes were accompanied with a reduction in serum and chondrocyte-derived TGF-β levels and increased levels of P-SMAD1/5. In WT mice, *Spon1* expression was observed as positive immunostaining in the epiphyseal cartilage region of long bones. We speculate that its inactivation primarily affects chondrocytes within this region, resulting in decreased secretion of TGF-β, which in turn leads to reduced TGF-β signaling in adjacent bone, and enhanced activation of SMAD 1/5 dependent osteogenic pathways.

Somewhat surprisingly, *Spon1* deletion did not cause any gross abnormalities in growth plate organization or significant impairment of endochondral development. This finding was unexpected given that we have previously shown F-spondin expression in hypertrophic cartilage and its regulation of chondrocyte terminal differentiation when added exogenously to cell and tibia explant cultures [7]. Moreover, chondrocyte cell cultures also exhibited reduced TGF-β levels and elevation of P-SMAD1/5, both of which have been shown to affect terminal differentiation [25]. The lack of pronounced defects in endochondral development may be attributed to compensatory effects by other TSP family members following *Spon1* deletion. In this regard, *Spon1* **^−^** ^/−^ mice share similarities in skeletal phenotype with *Tsp2* and *Tsp3* null mutants in that prenatal development appears normal, but mice exhibit bone accumulation during adulthood [26], [27]. For articular cartilage development and maintenance, our findings suggest that *Spon1* is also dispensable, evidenced by ‘normal’ cartilage thickness and morphology in KO mice up to 12 months of age. This finding was not surprising, as we have previously shown that *Spon1* is not expressed in normal articular cartilage, and present only after onset of osteoarthritis [14].

Analysis of femurs and tibiae by micro-CT revealed that the increased bone mass associated with *Spon1* deletion in adult mice varied with age. Bone volume (BV/TV) and trabecular number (Tb.N) increased in null mice at 3 months, but these differences were not significant until 6 months of age. By 12 months, the phenotype was still apparent in trabecular bone, but to a lesser degree, and no changes were observed in cortical bone. Of relevance to these findings, Glatt et al. demonstrated that C57Bl/6 mice undergo trabecular bone loss that is characterized by a rapid decline between 2 and 6 months and a more gradual decrease thereafter, while cortical bone remains relatively stable [28]. Consistent with these data, progressive bone loss was also evident in WT mice from our study which were analyzed within a similar time frame (3–12 months). Thus the variations among different age groups could reflect the impact of underlying bone loss and remodeling processes masking the *Spon1* **^−^** ^/−^ phenotype. Further analysis of aged mice would be needed to determine if *Spon1* **^−^** ^/−^ inactivation continues to slow age-related bone loss after 12 months.

Despite the high bone mass phenotype of adult *Spon1* **^−^** ^/−^ mice there have been no previous reports of F-spondin expression in adult bone, or isolated cell cultures of osteoblasts or osteoclasts. Likewise, we found either low or absent endogenous F-spondin levels in osteoclasts and osteoblasts of WT mice by QPCR. Gene expression profiling has identified significant upregulation of F-spondin in bone marrow cells of rats, following induction of osteoporosis [29]. This finding suggests an association of F-spondin with bone loss; however its role was not investigated further. While the mechanism underlying the increased bone phenotype was not directly determined in our study, we did not find any evidence of impaired bone resorption or osteoclast maturation in *Spon1* **^−^** ^/−^ mice, despite a localized increase in TRAP staining in femurs and tibia. A potential explanation for this discrepancy is that serum levels represent all bone, and osteoclast differentiation assays were determined from bone marrow extracts obtained from multiple locations. The increased TRAP staining, along with alkaline phosphatase and periostin may therefore reflect a localized increase in overall bone turnover within the growth plate region. A limitation of our study is that analyses were confined to femurs and tibiae only: therefore it is not known whether the bone phenotype also extends to vertebrae or intramembranous bones.

Since TGF-β regulates both synthetic and resorptive processes of bone development and homeostasis [20], extracellular matrix proteins that either, bind, activate or sequester the latent TGF-β complex are also likely to play an important role in regulating bone remodeling. We have previously reported that F-spondin increases active TGF-β1 levels in OA and hypertrophic cartilage [7], [8]. The current study showed further evidence of TGF-β regulation by *Spon1* and provides a possible mechanism for regulation of bone mass. In agreement with our previous *in vitro* observations, active TGF-β levels were reduced in cultured rib chondrocytes of KO mice. Somewhat unexpectedly, total (latent + active) TGF-β1 levels were also reduced, suggesting either down-regulation of a TGF-β autocrine loop, or inhibition of TGF-β synthesis levels via an alternate pathway. TGF-β levels were also significantly reduced in serum of KO mice, but not in other tissues that also showed endogenous expression of F-spondin mRNA in WT animals. These observations may reflect tissue specific variations in TGF-β regulatory proteins that promote its synthesis and activation. Further genetic approaches will be needed to clarify the role of serum and chondrocyte-derived TGF-β in *Spon1* regulation of bone metabolism.

Genetic deletion of TGF-β-binding ECM proteins LTBP-3, fibrillin-1, fibrillin-2, have also led to bone phenotypes in KO mice [30], [31]. In both cases, the authors speculate that defects in extracellular matrix assembly directly affect TGF-β bioavailability and subsequent regulation of bone mass. By comparing fibrillin-1 and fibrillin-2 mutant mice, Nistala et al. showed that reduced bone mass and impaired osteoblast maturation occur in response to elevated TGF-β when P-SMAD2/3 levels are increased relative to P-SMAD1/5 [31]. Our findings are similar in that *Spon1* **^−^** ^/−^ mice display the opposing trend; increased bone, decreased TGF-β and increased levels of P-SMAD1/5. Collectively, these observations suggest an inverse relationship between TGF-β levels and bone synthesis *in vivo*, via the differential activation of SMAD family transcription factors. Increased SMAD1/5 levels are compatible with increased bone mass, since both of these molecules have been shown to co-operate with RUNX-2 in promoting BMP-driven osteoblast differentiation [32]. Moreover, TGF-β has been shown to impair BMP-associated SMAD1/5/8 signaling and osteoblast differentiation *in vitro* [33], [34]. Thus, increased SMAD 1/5 signaling following a reduction in local or systemic TGF-β levels may offer a plausible mechanism for increased bone mass in *Spon1* null mice.
